# Editorial: Advancing the Frontiers of Non-Invasive Neuromodulation in Research and Clinical Practice

**DOI:** 10.3390/brainsci16020178

**Published:** 2026-01-31

**Authors:** Mehran Emadi Andani, Fatemeh Yavari

**Affiliations:** 1 Department of Neurosciences, Biomedicine and Movement Sciences, University of Verona, 37131 Verona, Italy; 2 Leibniz Research Centre for Working Environment and Human Factors (IfADo), Ardeystrasse 67, 44139 Dortmund, Germany

The clinical management of neurological and psychiatric disorders is currently witnessing a paradigm shift. We are moving from an era defined largely by systemic pharmacological interventions toward an era of circuit-specific modulation. Non-invasive brain stimulation (NIBS) techniques have matured from experimental tools into potent clinical modalities capable of inducing neuroplasticity and modulating pathological network oscillations [1]. This Special Issue, “Noninvasive Neuromodulation Applications in Research and Clinics,” captures this momentum through a collection of articles that traverse the spectrum of the field—from bibliometric trends and computational modeling to pilot trials and long-term clinical outcomes.

Parkinson’s Disease (PD) remains a primary frontier for neuromodulation. In this issue, Moshayedi et al. (2025) provide a critical synthesis of the current armamentarium, contrasting invasive Deep Brain Stimulation (DBS) with NIBS modalities like Transcranial Magnetic Stimulation (TMS) and transcranial Electrical Stimulation (tES) [2]. While DBS remains the standard for advanced and refractory cases [3], the authors highlight the safety profile of NIBS for managing milder symptoms. Bridging theory and practice, Mazzara et al. (2025) investigate transcranial random noise stimulation (tRNS) targeting the dorsolateral prefrontal cortex [4]. Their randomized, double-blind pilot study demonstrated that active tRNS improved executive function in PD patients, suggesting noise stimulation as a viable tool for cognitive rehabilitation.

However, the optimization of these stimulation parameters remains a substantial methodological challenge. Theoretical insights from computational modeling are essential for refining these protocols. In a series of related studies, Rouhollahi and colleagues demonstrated that robust feedback control systems—particularly those stimulating multiple basal ganglia targets simultaneously—can optimize therapeutic efficacy while minimizing energy delivery [5,6,7]. Their work on robust adaptive controllers [8] and backstepping controllers [5] highlights the importance of controllability and observability in biological models [9]. These computational principles are increasingly relevant as we seek to refine non-invasive protocols to match the efficacy of surgical interventions.

As the global burden of dementia continues to rise, the focus has shifted toward interventions that slow or stabilize disease progression. Chen et al. (2025) provide a bibliometric analysis of neuromodulation for language deficits in Alzheimer’s Disease (AD) [10], revealing a surge in research activity regarding rTMS and tDCS since 2021. Moving from research trends to clinical feasibility, Cont-Richter et al. (2025) present compelling long-term data on Transcranial Pulse Stimulation (TPS) [11]. In a one-year prospective study, they demonstrated that TPS is safe and capable of stabilizing cognitive scores in AD patients—a population that typically exhibits progressive decline. This aligns with accumulating evidence suggesting that preservation of synaptic plasticity is key to delaying neurodegeneration [12].

The scope of NIBS is also expanding rapidly into psychiatry. Du et al. (2025) reviewed the efficacy of Low-Intensity Focused Ultrasound (LIFU) for depression and anxiety [13]. Unlike superficial stimulation methods, LIFU offers a high spatial precision and deeper penetration, capable of modulating subcortical structures like the amygdala [14]. Their review indicates that LIFU can regulate neurotrophic factors like brain-derived neurotrophic factor (BDNF), positioning it as a promising intervention for treatment-resistant conditions. Furthermore, understanding physiological mechanisms is crucial. Mitsutake et al. (2025) contributed an exploratory study on Galvanic Vestibular Stimulation (GVS), shedding light on the neural mechanisms of vestibular-related postural control through analysis of the soleus H-reflex [15].

The integration of these diverse methodologies—from the molecular mechanisms of ultrasound to the network effects of magnetic stimulation—signals a bright future. Emerging technologies such as temporal interference stimulation [16] and personalized “digital twins” promise to further enhance targeting precision. As highlighted by the contributions in this Special Issue, the field is steadily moving toward personalized, multimodal stimulation protocols that maximize patient outcomes while minimizing invasiveness.

## Conclusions

In conclusion, the contributions to this Special Issue collectively illustrate a transformative era in the management of neurological and psychiatric disorders. As the field advances from elucidating fundamental mechanisms—such as the physiological responses to vestibular stimulation—to validating long-term clinical efficacy in complex conditions like Alzheimer’s and Parkinson’s disease, the potential of non-invasive neuromodulation becomes increasingly evident. The synthesis of rigorous clinical trials with insights from computational modeling and control theory suggests that the future of therapy lies in precision, adaptability, and personalization. By optimizing stimulation parameters to target specific neural circuits while minimizing energy delivery and side effects, researchers are paving the way for safer, more effective interventions. Ultimately, the continued convergence of engineering, neuroscience, and clinical practice promises to refine these powerful tools, offering renewed hope and improved quality of life for patients worldwide.
